# Supplementary material for: Essential Oils as Nematicides in Plant Protection—A Review
Source: Plants (Basel). 2023 Mar 22;12(6):1418. doi: 10.3390/plants12061418 (PMC10058003; doi:10.3390/plants12061418)
Supplement: Supplementary file 1 [file plants-12-01418-s001.zip › plants-2280642-supplementary.pdf]

SUPPLEMENTARY MATERIAL

Table S1. Nematode classification (family, genus, and species) of the species studied in documents downloaded for the systematic analysis. The classification includes the putative level, c-p, and feeding group of nematodes studied. The classification data have been taken from the Nemaplex database (http://nemaplex.ucdavis.edu), a free virtual Encyclopedia on soil and plant nematodes.

Nematode family	Nematode genus	Nematode species	Putative Feeding	c-p group	Feeding group	References
Anguinidae	*Ditylenchus*	*D. dipsaci*	plant feeder	2	1	Zouhar et al. (2009)
Aphelenchoididae	*Bursaphelenchus*	*B. xylophilus*	plant feeder	2	1	Park et al. (2005); Kong et al. (2007); Park et al. 2007; Elbadri et al. (2008); Kim et al. (2008); Kong et al. (2009); Barbosa et al. 2010; Faria et al. (2013)
Criconematidae	*Criconemella* spp.		plant feeder	3	1	Abd-Elgawa and Omer (1995)
Heteroderidae	*Meloidogyne* spp.		plant feeder	3	1	Madalosso et al. (2017); Ntalli et al. (2020)
Heteroderidae	*Meloidogyne*	*M. incognita*	plant feeder	3	1	Pandey et al. 2000; Ibrahim et al. (2006); Romero et al. (2006); Meyer et al. (2008); Abu Elyosur (2009); Cetintas and Jarbas (2010); Ntalli et al. (2010); Ntalli et al. (2011); Caboni et al. (2013); Quin Lin et al. (2013); Laquale et al. (2015); Avato et al. (2017); Sendi and Khosravi (2017); Laquale et al. (2018); Jardim et al. (2018); Barros et al. (2019); Eloh et al. (2019); Kalaiselvia et al. (2019); D'Addabbo et al. (2020); Jardim et al. (2020); Pardavella et al. (2020); Dutta et al. (2021); Kundu et al. (2021)
Heteroderidae	*Meloidogyne*	*M. javanica*	plant feeder	3	1	Oka et al. (2000); Onifade et al. (2008); Ntalli et al. (2011); Sosa et al. (2012); Mattei et al. (2013); Santana et al. (2014); Sousa et al. (2015); Andres et al. (2017); Julio et al. (2017); Massuh et al. (2017); Borges et al. (2018); Navarro-Rocha et al. (2020); Pardavella et al. (2020); Basaid et al. (2021); Galitseo et al. (2022)

Heteroderidae	*Meloidogyne*	*M. chitwoodi*	plant feeder	3	1	Faria et al. (2016)
Heteroderidae	*Meloidogyne*	*M. arenaria*	plant feeder	3	1	Walker and Melin (1996); Caboni et al. (2014)
Heteroderidae	*Meloidogyne*	*M. artiellia*	plant feeder	3	1	Perez et al. (2003)
Heteroderidae	*Meloidogyne*	*M. graminicola*	plant feeder	3	1	Chavan et al. (2019)
Hoplolaimidae	*Rotylenchulus*	*R. reniformis*	plant feeders	3	1	Abd-Elgawa and Omer (1995)
Hoplolaimidae	*Hoplolaimus* spp.		plant feeder	3	1	Abd-Elgawa and Omer (1995)
Panagrolaimidae	*Panagrolaimus* spp.		bacterial feeder	1	3	Oro et al. (2020)
Pratylenchidae	*Pratylenchus*	*P. vulnus*	plant feeder	3	1	Avato et al. (2017); Laquale et al. (2018)
Pratylenchidae	*Nacobbus*	*N. aberrans*	plant feeder	3	1	Sosa et al. (2020)
Pratylenchidae	*Pratylenchus*	*P. brachyurus*	plant feeder	3	1	Mattei et al. (2013)
Rhabditidae	*Caenorhabditis*	*C. elegans*	bacterial feeder	3	1	Satyal et al. (2012); Kalaiselvia et al. (2019)
Longidoridae	*Xiphinema*	*X. index*	plant feeder	5	1	Avato et al. (2021)
